# New York Letter

**Published:** 1902-07

**Authors:** 


### Domestic Correspondence.

### NEW YORK LETTER.

To the Editor:

Sir,—The summer months being upon us, our different dental societies here, feeling the need of rest after their long season's work, have all, with the exception of The New York Institute of Stomatology, adjourned until the autumn. A regular monthly meeting of that society met on May 6, at the office of Dr. Geo. S. Allan, and the subject was one destined to assume great proportions ere many months or years have passed, it being the dentist's relation or association to general hospitals.

Dr. Flanagan, of Springfield, Mass., was the essayist of the evening, the title of the paper being "The Hospital's Need of a Dental Staff." Dr. Flanagan discoursed at some length upon the dentist's relation and value in hospital service, maintaining that in many instances his services and special knowledge would be of inestimable value in materially assisting the general surgeon in the treatment of diseased conditions with which, by virtue of our vast experience, we have been familiar.

Papers by Dr. J. Leon Williams, of London, and Dr. Barnes, of Cleveland, sent by these gentlemen, were also read. Dr. Newton, of Montclair, a physician, being among those present, also expressed his views. All these spoke favorably of dentists working in harmony with the general medical profession for ameliorating the sufferings of the poor in hospital work.

In opening the discussion Dr. Dawbarn, who, by the way, was instrumental in having two dentists appointed on the staff of the largest hospital in New York,—the City Hospital,—spoke at some length upon the dentist's relation to the medical profession, maintaining that the dentist's relation should be akin to that of the laryngologist or any other specialist in medicine or surgery.

Dr. Gillett pointed out the possibilities in the treatment and advice that could be given to children in hospital practice which might enable them to give the proper attention to their teeth in after years, and also considered this important subject from other aspects.

Dr. J. Adams Bishop, of New York, presented a large number of interdental splints which he had made and inserted for fractured maxillæ in hospital work, in which, by the way, this distinguished authority has accomplished much, helping many unfortunate sufferers by devoting for a number of years a certain amount of his time and unquestioned skill in hospital service.

Professor Brackett, of Newport, spoke at some length upon the relation of the dentist to that of the surgeon or physician, and was listened to with great interest; and after taking a favorable view of the subject, expressed his kindly appreciation for the great interest which the general medical profession had at last taken in us, they recognizing the importance of hearty co-operation in this field. Many others also spoke, and after taking suitable action on the loss of Dr. Benjamin Lord, their honored first president, the meeting adjourned.

Some time ago I wrote in regard to an address by Dr. T. W. Brophy before a medical society, in which I was under the impression that he stated that out of six hundred and fifty cases operated upon for cleft palate, two hundred and fifty had been successful, where I should have stated that of six hundred and fifty cases operated upon, two hundred and fifty cases were performed by transfixing the bones in young children.

"LOCHINVAR."

